# Temporal Dynamics of ‘*Ca.* Phytoplasma mali’ Load in the Insect Vector *Cacopsylla melanoneura*

**DOI:** 10.3390/insects11090592

**Published:** 2020-09-03

**Authors:** Valentina Candian, Monia Monti, Rosemarie Tedeschi

**Affiliations:** Dipartimento di Scienze Agrarie, Forestali e Alimentari (DISAFA), University of Torino, Largo P. Braccini 2, 10095 Grugliasco (TO), Italy; valentina.candian@unito.it (V.C.); monia.monti1983@gmail.com (M.M.)

**Keywords:** psyllid vector, acquisition, latency, vector competence, phytoplasma titer, apple proliferation, epidemiology, Northwest Italy

## Abstract

**Simple Summary:**

*Cacopsylla melanoneura* is a psyllid vector of the phytoplasma ‘*Candidatus* Phytoplasma mali’, the causal agent of apple proliferation. It spends part of the year in apple orchards, then moves to conifers for aestivation and overwintering. The aim of this work was to analyze the quantity of phytoplasma inside the insect body throughout the year, during different life stages, in order to assess the relative acquisition efficiency and the vector infective risk. The phytoplasma was detected in all the life stages. The phytoplasma load significantly increased during the period spent on conifers, but in many cases also nymphs and newly emerged adults contained a sufficient quantity of phytoplasma to consider these stages infective. The obtained results allow us to optimize control strategies against the vector and thus against the spread of the disease.

**Abstract:**

The transmission of phytoplasmas is the result of an intricate interplay involving pathogens, insect vectors and host plants. Knowledge of the vector’s competence during its lifespan allows us to define more sustainable well-timed control strategies targeted towards the most worrisome life stages. We investigated the temporal dynamics of ‘*Candidatus* Phytoplasma mali’ load in *Cacopsylla melanoneura* in the different developmental stages in Northwest Italy. The phytoplasma load in the vector was evaluated in overwintering adults, nymphs and newly emerged adults after different acquisition access periods. Moreover, we followed the multiplication of the phytoplasma during the aestivation and the overwintering period on conifers. Our results confirmed the ability of remigrants to retain the phytoplasma until the end of winter. We also highlighted the high acquisition efficiency and vector competence, based on phytoplasma load, of nymphs and newly emerged adults. Therefore, particular attention should be paid to the management of overwintered *C. melanoneura* as soon as they return to the orchards, but also to newly emerged adults, particularly in orchards with a high infection rate and when the migration to conifers is delayed.

## 1. Introduction

Phytoplasmas are wall-less phytopathogenic prokaryotes transmitted by phloem-sucking insects in a persistent propagative manner and are associated with several diseases in both cultivated and wild plants [1]. The transmission of phytoplasmas is the result of an intricate interplay involving pathogens, insect vectors and host plants [2].

Focusing on the vector, the complete transmission process is commonly divided into three phases: the acquisition period (the length of time a vector must feed on an infected plant to acquire sufficient phytoplasma to become infective), the latency period (the time interval in which phytoplasmas invade the insect body via the hemolymph, multiply and reach the salivary glands, leading the insect to become infective) and the inoculation period (the time required to transmit the phtoplasma to a healthy plant during feeding activity) [3,4,5]. The length of these phases (acquisition, latency, inoculation)—commonly ranging from a few hours to several days for the acquisition period, from 12 days to well over a month for the latency period and about a few hours for the inoculation period—are strongly influenced by several factors, such as the phytoplasma species/strain, the phytoplasma titer, the host plant, the vector species, gender, age and environmental factors such as temperature [6,7,8,9,10,11,12,13,14,15]. 

Knowledge of the details of the transmission processes is of paramount importance for the management of the spread of the diseases, which is in most cases based on the control of the insect vectors. As a consequence, control strategies should be targeted towards the key period of the vectors, considering not only their abundance, but also their vector competence and efficiency during the season, in relation to phytoplasma load. It is therefore clear that great differences occur in different pathosystems. Although the study of these parameters can be quite easy in the case of monophagous insects with a simple biological cycle, it can be more complicated in the case of oligophagous or polyphagous vectors with more complex biological cycles. Such is the case for most of the psyllid vectors of phytoplasmas that spend a great part of the year on conifers as shelter plants for aestivation and overwintering, even kilometers away from apple orchards [16,17]. For a long time, this particular biological cycle hampered the constant monitoring of these vectors and prevented the detailed analysis of phytoplasma load inside them throughout the year. Although some attempts were made to reproduce the whole biological cycle in the laboratory [18], an accurate study of the phytoplasma multiplication in psyllids can be carried out only by isolating the vectors on the shelter plants in nature and testing them throughout the year, as was done by Thébaud et al. [19] with *Cacopsylla pruni* (Scopoli) (Homoptera: Psyllidae). Phytoplasma multiplication during aestivation and overwintering on conifers is indeed a phenomenon regulated by complex interactions of biotic and abiotic factors, such as temperature, relative humidity and air pressure due to altitude, which cannot accurately be reproduced in the laboratory.

Moreover, the vector competence and efficiency can be strongly influenced, even within the same species, by different associations of vector populations and phytoplasma strains occurring in different geographical areas, as highlighted for the psyllid *Cacopsylla melanoneura* Förster (Homoptera: Psyllidae), vector of ‘*Candidatus* Phytoplasma mali’, the causal agent of apple proliferation disease [20]. In particular, there is a strict co-occurrence of AT-1-associated subtypes of ‘*Ca.* P. mali’ and *C. melanoneura* in Northwest Italy, where *C. melanoneura* is considered to be the most important vector of this phytoplasma [21], whereas in other regions, where *C. melanonera* is considered an ineffective or a low efficient vector, ‘*Ca.* P. mali’ subtypes AT-2 [20,22,23] or AP [22,24,25] are present.

*Cacopsylla melanoneura* starts to colonize apple trees at the end of January and reaches peak density around mid-March [26]. At the end of May, the newly emerged adults rapidly move to conifers, which they use as shelter plants for aestivation and overwintering [27]. To date, in the regions were *C. melanoneura* has a relevant role in the transmission of ‘*Ca*. P. mali’, transmission trials and molecular analyses carried out with specimens collected on apple plants have highlighted the crucial role of overwintered individuals in comparison to newly emerged adults due to a higher percentage of ‘*Ca.* P. mali’-positive individuals (3.6% vs. 0.8%) and a longer period spent in apple orchards (14.6 vs. 6 weeks) [26,28]. On the contrary, several doubts remain regarding the ability of nymphs and newly emerged adults to reach the latency period before migration to conifers, due to a lower efficiency in transmitting the phytoplasma [29]. The discovery, in recent years, of sites colonized by *C. melanoneura* during aestivation and overwintering in Northwest Italy, after the migration from apple orchards, has made it possible to follow the entire life cycle of the vector, opening up new perspectives on the study of phytoplasma vector relationships during the entire year and thus on the epidemiology of apple proliferation [27].

The aim of the present work was to investigate the temporal dynamics of ‘*Ca.* P. mali’ load in the insect vector *C. melanoneura* in order to highlight the acquisition competence and the infective risk of the different developmental stages during the whole year in one region (Aosta Valley, Northwest Italy), in which the role of this vector in the spread of Apple proliferation phytoplasma (APP) has been proved to be crucial [26,28,29].

## 2. Materials and Methods

### 2.1. Experimental Sites and Insect Sampling

All the insects used in the experiments originated from *C. melanoneura* overwintered adults collected using the beating tray sampling method in commercial apple orchards located in the Aosta Valley region of Northwest Italy at an altitude ranging from 600 to 820 m a.s.l. from February to March. In order to avoid as much as possible the collection of already naturally infected insects, psyllids were sampled in orchards with a low incidence of apple proliferation in the previous year and from asymptomatic plants. Furthermore, a portion of the overwintered adults (180 individuals) were directly tested by molecular analysis to assess the natural infection rate and the phytoplasma load, and the others were designated for the experimental trials.

Aestivation and overwintering trials were carried out in a *Picea abies* L. forest at an altitude ranging from 1350 to 1550 m a.s.l. in the Aosta Valley region, around 2 km away from the closest apple orchard as the crow flies. This site was chosen according to previous studies that identified it as a suitable area for *C. melanoneura* aestivation and overwintering [27].

### 2.2. Insect Rearing and ‘Ca. Phytoplasma mali’ Acquisition Trials 

Field-collected overwintered adults were transferred under insect rearing tents (75 × 75 × 115 cm) (BugDorm, MegaView, Taiwan) on three 6–8-year-old ‘*Ca*. P. mali’ (strain AT-1)-infected apple plants (previously tested for the presence of the phytoplasma by real-time PCR) at the Department of Agriculture, Forest and Food Science, DISAFA; University of Torino, Grugliasco, Italy. Infected plants were kept short, never higher than 110 cm. Psyllid rearing on infected plants was carried out for phytoplasma-controlled acquisition trials and thus maintained, after the egg laying, until the emergence of the new adults. These rearing plants were maintained outdoors in natural conditions.

In order to evaluate the phytoplasma acquisition efficiency by *C. melanoneura* during its different life stages, some of the overwintered adults (331 specimens) reared on infected apple plants were removed after 1, 2, 10 and 20 days of the acquisition access period (AAP) and were quickly frozen at −20 °C until phytoplasma titer analysis. Likewise, 102 nymphs (42 from the first to the second instar and 60 from the third to the fifth instar) and 83 newly emerged individuals were collected from ‘*Ca*. P. mali’-infected plants and were quickly frozen at −20 °C before being processed for phytoplasma titer analysis. Nymphs and newly emerged specimens were collected after an acquisition access period of about 10–20 and 30 days, respectively.

### 2.3. Dynamics of ‘Ca. Phytoplasma mali’ Load During C. melanoneura Aestivation and Overwintering

**Figure 1:**
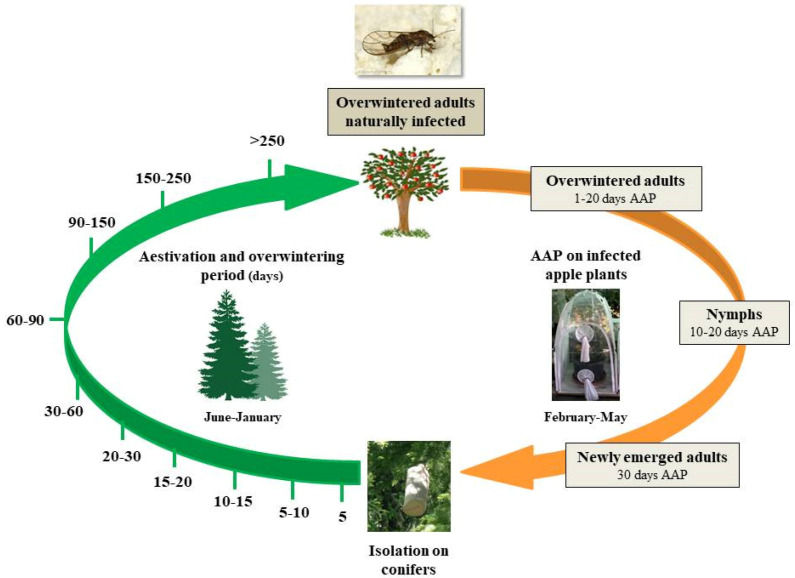
Experimental design followed to evaluate phytoplasma acquisition efficiency by *Cacopsylla melanoneura* during its different life stages (orange arrow) and the dynamics of ‘*Candidatus* Phytoplasma mali’ load during *C. melanoneura* aestivation and overwintering on conifers (green arrow). AAP = acquisition access period; APP = apple proliferation phytoplasma.

Immediately after emergence, new adults were periodically transferred to conifers in order to follow the multiplication of the phytoplasma during the aestivation and overwintering period. Batches of 20 newly emerged individuals were isolated inside cylindrical cages (length 29 cm; diameter 10 cm) made with a plastic net and covered with a strong nylon sock. Each cage was put at the extremity of *P. abies* branches and psyllids were introduced inside the cages. A total of 1000 specimens were isolated in 50 cages. After 5, 10, 15, 20, 30, 60, 90, 150, 250 and >250 days spent on conifers, groups of cages were removed, brought to the laboratory and living specimens were counted and preserved at −20 °C before the analysis of phytoplasma titer (Figure 1).

### 2.4. DNA Extraction and ‘Ca. Phytoplasma mali’ Quantification via RT-qPCR

Total DNA was extracted from single insects using a CTAB-based protocol, as described in Bertin and Bosco [30], which has already been applied to psyllids [26]. Single psyllids were grounded in a 1.5 mL microtube with 500 µL of prewarmed (60 °C) 2% CTAB buffer (2% wt:vol cethyltrimethyl-ammonium-bromide buffer, 1.4 M NaCl, 20 mM EDTA pH 8.0, 100 mM Tris-HCl pH 8.0). After clarification with chloroform-isoamyl alcohol (24:1, vol:vol) (Merck KGaA, Darmstadt, Germany), the DNA was precipitated from the aqueous phase with 1 volume of isopropanol (Merck KGaA, Darmstadt, Germany) and microcentrifuged. The DNA pellet was washed with 70% ethanol, dried and resuspended in 1× TE buffer. DNA quality and quantity were assessed by a NanoDrop Spectrophotometer (Thermo Fisher Scientific, Wilmington, DE, USA). As mentioned above, before being used in the trials, phytoplasma presence in the experimental plants was confirmed. Plant DNA was isolated from 100 mg (wet weight) of phloem tissue previously ground with liquid nitrogen in a sterile mortar, using the QIAGEN DNeasy Plant Mini Kit (Qiagen, Hilden, Germany) and following the manufacturer’s instructions. DNA was eluted in 100 µL of elution buffer and kept at −20 °C until used.

The real-time PCR procedure described in Monti et al. [31] was followed in order to quantify the apple proliferation phytoplasma in all the *C. melanoneura* individuals used in the trials. Briefly, two absolute quantitative real-time PCRs, having different targets, were used. In the first amplification the rpAP15f-mod/rpAP15r3 primer pair (5’-TGCTGAAGCTAATTTGGC-3’/5’-CCCATGAATATTAACCTCCT-3’) [32], which amplifies a specific fragment of 238 bp located in the variable region of the ribosomal protein (rp) *rplV* gene (rpl22) of ‘*Ca*. P. mali’, was used. In order to normalize quantification data, a second real-time PCR, which amplifies a 98 bp region of the insect 18S rDNA gene, was run using the primer pair MqFw/MqRv (5’-AACGGCTACCACATCCAAGG-3’/5’-GCCTCGGATGAGTCCCG-3’) [33]. The real-time PCRs were carried out in a 25-µL volume, comprising the following components: 12.5 mL of SsoFast^TM^ EvaGreen^®^ Supermix 2× (Bio-Rad, Hercules, CA, USA) (containing dNTPs, Sso7d fusion polymerase, MgCl_2_, EvaGreen^®^ dye and stabilizers), 2.5 µL of each primer (3 µM), 6.5 µL of Milli-Q water and 1 µL of DNA templates. All standards and samples were run in triplicate. An additional sample, containing water instead of DNA, was added to each plate in triplicate as a DNA-free negative control. The reaction was conducted in a DNA Engine Opticon^TM^ System (Bio-Rad, Hercules, CA, USA). For the amplification of ‘*Ca*. P. mali’ the following thermal conditions were used: incubation step at 95 °C for 2 min, 40 cycles of amplification at 94 °C for 15 s, 56 °C for 15 s, 72 °C for 20 s and a final extension step at 72 °C for 8 min. On the contrary, for the quantification of 18S rDNA, the following thermal protocol was used: incubation step at 94 °C for 3 min, 37 cycles of amplification at 94 °C for 45 s, 65 °C for 1 min. Two melting curve profiles, the first ramp from 65 °C to 95 °C at 0.2 °C/s and the second one from 65 °C to 94 °C at 0.5 °C/s, were run. Quantification was made by interpolation of the C_T_ of each sample against standard curves prepared from serial dilutions of plasmids containing *rplV-rpsC* and a portion of the 18S rDNA gene, respectively, followed by specific calculations in order to normalize ‘*Ca*. P. mali’ quantification data. Analyses of melting curves generated from each assay were also carried out for each template in order to detect possible non-specific products. The ‘*Ca*. P. mali’ titer in insects was expressed as genome units (GU) of phytoplasma per picogram (pg) of individual insect 18S rDNA. Plant DNAs were analyzed by an absolute quantitative real-time PCR, using the primers rpAP15f-mod/rpAP15r3, as described for the insects.

### 2.5. Statistical Analyses

Data analyses were performed using SPSS v26.0 (SPSS Inc., Chicago, IL, USA) and outcomes were considered significant at *p* < 0.05. Statistical analysis was performed taking into account only the positive specimens. The ‘*Ca*. P. mali’ titers of the infected *C. melanoneura* instars were compared using a generalized linear model (GLM) with a normal distribution, identity link and Bonferroni correction.

## 3. Results

### 3.1. Natural Infection and Controlled Acquisition of ‘Ca. Phytoplasma mali’

The natural infection rate of *C. melanoneura* overwintered adults tested directly after field collection was 1.67% (three positive out of 180 tested specimens) with a mean phytoplasma concentration of 2.04 × 10^3^ ± 5.44 × 10^2^ GU/pg insect 18S rDNA. Phytoplasma loads equal to 2.01 × 10^8^, 1.06 × 10^8^ and 7.92 × 10^6^ phytoplasma copies/sample (100 mg plant tissues) were observed at the beginning of the trials in the three infected apple plants used for the AAP trials.

**Figure 2:**
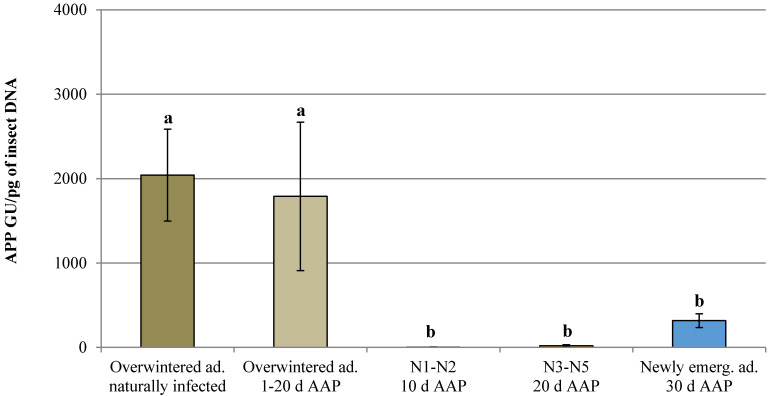
Quantification of apple proliferation phytoplasma (APP) titer expressed as genomic units (GU) of phytoplasma/pg of insect DNA in naturally infected overwintered adults and in experimentally infected overwintered adults, 1^st^–2^nd^ instar nymphs (N1–N2), 3^rd^–5^th^ instar nymphs (N3–N5) and newly emerged adults after different days of acquisition access period (AAP). Bars followed by different letters are significantly different (GLM: *p* < 0.05).

The number of infected specimens for each life stage and AAP observed in the trials is reported in Table 1. Unfortunately, only one infected overwintered adult was recorded after 2 and 20 days of AAP, so in order to statistically analyze the data, all the infected specimens recorded after 1, 2, 10 and 20 days of AAP were clustered in a unique group (overwintered adults 1–20 days of AAP). ‘*Candidatus* P. mali’ was detected in all *C. melanoneura* developmental stages and a higher acquisition efficiency was observed in the 3^rd^–5^th^ instar nymphs and newly emerged adults (infection rate of 48.33% and 42.17% respectively). Significant differences for the phytoplasma titer were observed among the life stages before the transfer to conifers (GLM: Wald Chi-Squared Test = 41.678; df = 4; *p* = 0.001) with higher values recorded in the overwintered adults, both naturally and experimentally infected (Figure 2). In the experimentally infected overwintered adults, a quite high standard error was recorded, due to three specimens that contained 7.65 × 10^3^; 2.14 × 10^3^ and 1.96 × 10^3^ GU/pg insect 18S rDNA, respectively, whereas in the others the phytoplasma titer ranged around 5.14 × 10^2^ GU/pg insect 18S rDNA. These high phytoplasma titers were all recorded in psyllids analyzed after 10 days of AAP. The phytoplasma titer of six newly emerged adults out of 35 positive individuals analyzed was higher than 1.00 × 10^3^ GU/pg insect 18S rDNA (1.74 × 10^3^, 1.09 × 10^3^, 1.05 × 10^3^, 1.13 × 10^3^, 1.43 × 10^3^ and 1.10 × 10^3^ GU/pg insect 18S rDNA), whereas in the others the phytoplasma titer ranged around 1.23 × 10^2^ GU/pg insect 18S rDNA. 

### 3.2. Dynamics of ‘Ca. Phytoplasma mali’ Load During the Aestivation and Overwintering Period

In total, 75.4% (754 out of 1000) of the caged psyllids survived and were analyzed for the phytoplasma titer. The infection rate and the phytoplasma titer recorded at different time points are reported in Table 2. Infected specimens were observed in all the tested time intervals except for the 5-day stay on conifers, and an infection rate above 50% was recorded after 5–10; 15–20 and more than 150 days on shelter plants. Some quite high phytoplasma titers were observed at different time points, especially within 30 days, whereas later values were more closely related. In particular, a titer up to 13 times higher was recorded after 5–10 days on conifers (9.34 × 10^2^ GU/pg insect 18S rDNA), whereas in the others of the same category the phytoplasma titer ranged around 7.31 × 10 GU/pg insect 18S rDNA. In two psyllids out of 28 individuals analyzed after 10–15 days on conifers, the phytoplasma titer was up to 33 times higher (9.52 × 10^2^ and 1.23 × 10^3^ GU/pg insect 18S rDNA), whereas in the others the phytoplasma titer ranged around 3.71×10 GU/pg insect 18S rDNA. Finally, the phytoplasma titer of four specimens analyzed after 20–30 days on conifers was up to 19 times higher (1.81 × 10^3^; 1.24 × 10^3^; 1.38 × 10^3^ and 2.24 × 10^3^ GU/pg insect 18S rDNA), whereas in the others the pathogen load ranged around 1.20 × 10^2^ GU/pg insect 18S rDNA.

**Figure 3:**
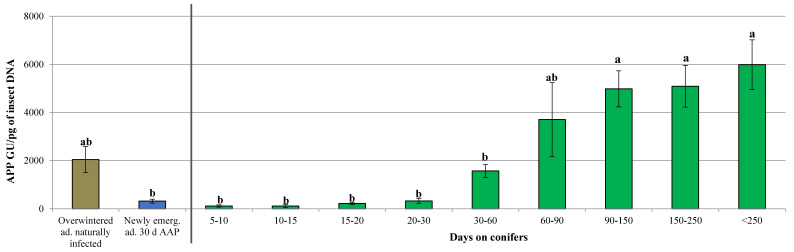
Quantification of apple proliferation phytoplasma (APP) titer expressed as genomic units (GU) of phytoplasma/pg of insect DNA in naturally infected overwintered adults and in newly emerged adults after a 30-day acquisition access period (AAP) and after further days spent on conifers. The groups that spent a controlled period on conifers are separated from the others by a vertical line. Bars followed by different letters are significantly different (GLM: *p* < 0.05).

The phytoplasma titers of naturally infected overwintered adults and of newly emerged individuals were also included in the statistical analysis to better evaluate the phytoplasma multiplication during the aestivation and the overwintering period. Significant differences for the phytoplasma titer were observed between the different tested groups (GLM: Wald Chi-Squared Test = 117.372; df = 8; *p* = 0.000) (Figure 3). An increase of the phytoplasma load proportional to the dwell time of the insect on conifers was recorded, with the higher titers occurring from 60 days onwards.

## 4. Discussion 

The assessment of phytoplasma acquisition efficiency and accumulation in insect vectors, as well as the potential infectivity of the insects at different life stages, based on the pathogen load, is fundamental in order to better understand the epidemiology of the diseases and to optimize the control strategies against the vectors and, as a consequence, the spread of the diseases. Two psyllid vectors of ‘*Ca*. P. mali’ are acknowledged, but in Northwest Italy only *C. melanoneura* has been reported [26,28,29]. In relation to its complex life cycle, the knowledge of the vector competence at the different life stages, and in specific moments within the period spent on apple trees allows us to define more sustainable well-timed control strategies targeted towards the most worrisome life stages.

Previous studies, based on molecular detection of the phytoplasma in the first overwintering adults collected in the apple orchards at the end of winter [28], already suggested the possibility that overwintering adults maintain the phytoplasma acquired in the previous season, whereas controlled transmission trials revealed that all stages are able to transmit the phytoplasma even with a very low percentage, in the case of nymphs and newly emerged adults [29]. However, no data were available regarding the dynamics of phytoplasma load in the vectors throughout the year. To our knowledge the present work is the first study that has analyzed the phytoplasma titer during the entire lifespan of *C. melanoneura*, producing useful results about the infective risk of the psyllid in different times of the year and confirming previous results on phytoplasma retention.

We started all the experiments with field-collected overwintered adults, being aware that these individuals might not be considered healthy even if sampled on asymptomatic plants. The natural infection rate was found to be very low (1.67%) and thus the probability to handle ‘*Ca*. P. mali’ positive specimens was very low as well. We also performed real-time qPCR on field-collected overwintered adults in order to compare their phytoplasma titers with the ones obtained after a controlled AAP, and eventually to highlight large differences ascribable to a natural infection.

Actually, in field-collected adults the phytoplasma titer was almost always 10 to 100 times higher than in the overwintered adults subjected to controlled acquisition trials, except for three individuals that were supposedly already infected before the AAP. In all the other cases, our results suggest the possibility that remigrants may still be able to acquire the phytoplasma, even at a low rate and in an uneven pattern compared with the AAP timing. Nil or one infected psyllids were recorded after 1, 2 or 20 days of AAP. This may be due to the short AAP (1 day), to the insect phytoplasma load that could be below the real-time PCR detection limit or to a reduced acquisition and retention efficiency in older overwintered adults.

As shown by RT-qPCR, first and second instar nymphs acquired ‘*Ca.* P. mali’, but at a very low percentage (7.14%) and, at the same time, in low quantities compared to the further stages. More concerning for their high phytoplasma content and infection rate are the 3^rd^–5^th^ instar nymphs and the newly emerged individuals that spent a longer time on infected plants. Our results confirmed the higher acquisition efficiency of immature stages, as already reported for several other phytoplasma vectors [5], whereas a significantly lower phytoplasma titer was recorded both in nymphs (3^rd^–5^th^ instar), and in newly emerged adults, compared with the naturally infected overwintered adults. This may suggest, as already reported by Thébaud et al. [19], that a longer latency period is required for immature psyllids to become infectious compared to other insect vectors.

More evidence confirming this hypothesis comes from the results obtained during the aestivation and overwintering period on conifers. Despite the reduction of the phytoplasma multiplication rate due to the decreasing of metabolic activity during the diapause, suggested by Oppedisano et al. [34], in our trials a constant increase of the ‘*Ca*. P. mali’ load was observed during the permanence on shelter plants. In addition, we demonstrated that the infectivity persists in *C. melanoneura* from spring to the following winter without an intermediate reacquisition, as already observed for *C. pruni*, the vector of ‘*Candidatus* Phytoplasma prunorum’ [19]. A slight decrease of the phytoplasma titer was initially observed just after the transfer of the newly emerged adults on conifers until a 15-day permanence on the shelter plants. We suggest that this phytoplasma titer loss could be attributable to the excretion of phytoplasma cells, ingested by the psyllids after feeding on infected apple plants, but which has not yet passed through the midgut and basement membrane before the shift to *P. abies*, where minimum feeding activity is supposed to occur [27]. A general relevant increase of the phytoplasma titer occurs proportionally after at least 15 days, suggesting the beginning of pathogen multiplication in the vector body, whereas a sort of plateau seems to be reached after 60 days spent on conifers.

After more than 60 days of permanence on conifers, *C. melanoneura* adults reached a phytoplasma load much higher compared to the naturally infected overwintered specimens. According to what was previously reported for Cicadellidae [35], it is possible to assume that insects with a phytoplasma load higher than 2.00 × 10^4^ GU/ng insect 18S rDNA could be infective. In our trials, this value occurred in all the psyllids after more than a 60-day stay on conifers, when phytoplasma titers ranged between 5.74 × 10 and 2.42 × 10^4^ GU/pg insect 18S rDNA (corresponding to 5.74 × 10^4^ and 2.42 × 10^7^ GU/ng insect 18S rDNA, respectively). However, some newly emerged adults reached this value in the first 60 days: 50%, 39%, 75%, 53% and 81% of the tested psyllids achieved a phytoplasma titer higher than 2.00 × 10 GU/pg insect 18S rDNA (corresponding to 2.00 × 10^4^ GU/ng insect 18S rDNA) after 10, 15, 20, 30 and 60 days spent on conifers, respectively. According to these results, newly emerged adults may transmit ‘*Ca.* P. mali’ from a few days after emergence. However, a few considerations need to be taken into account in this regard.

First, our experimental conditions slightly deviate from field conditions. The psyllid used in our trials constantly fed on infected plants from egg hatching to the emergence of the adults (about 30 days). The phytoplasma titer of test plants was certainly higher than the ones in the apple orchards, where trees could be healthy or larger in size. These conditions could present a higher dilution and an uneven distribution of the phytoplasma in the phloem. Only in the case in which eggs are laid on infected plants do nymphs constantly feed on infected plants, due to their low mobility until the adult phase, which increases the probability of transmission of ‘*Ca.* P. mali’. This situation is highly likely in orchards with a high infection rate. Moreover, newly emerged adults tend to rapidly migrate to shelter plants, so their impact on the spread of the disease in the orchards can be worrisome when their permanence on apple trees is prolonged, for instance, due to cold springs or unfavourable conditions for migration.

After the AAP, the phytoplasma load of a few newly emerged individuals was even higher (six values out of 35), compared to the one recorded in naturally infected overwintered adults. Even if the possibility of a transovarial transmission in *C. melanoneura* has never been demonstrated [36], this possibility should not be completely excluded, because it could explain these findings.

Our results differed from a previous Italian study [34] in which a very low percentage of infected individuals were observed after a controlled acquisition trial and which also led to a lower transmission efficiency compared with the congeneric *Cacopsylla picta* Förster (Homoptera: Psyllidae). The phytoplasma load seemed to be much lower, even if not easily comparable to our results. These differences confirm the existence of variability in vector competence between populations of *C. melanoneura* from different geographic areas. Differences in acquisition efficiency may be also be linked to the host plants and the pathogen strain AT-2 used in Oppedisano et al. [34]. The AT-2 strain of ‘*Ca*. P. mali’ is not as common in our region, and therefore we chose the AT-1 strain because it is representative of the typical pathogen encountered by *C. melanoneura* in apple orchards in Northwest Italy.

Although the trend of phytoplasma accumulation in the psyllids during aestivation and overwintering clearly demonstrates the achievement of a sort of saturation (after 90–150 days), this did not happen in all the individuals. If we look at the single values, we can see that there are specimens with only a few hundred phytoplasma GU even after > 250 days. It is likely that the quantity of phytoplasmas acquired during the first stages has a role, and only when a relatively “high” phytoplasma titer has been acquired (a sort of threshold) can the saturation point be reached. Alternatively, some unknown barriers may occur during the development. Further research is needed to clarify these aspects.

In light of the results obtained in the present study, we can confirm that particular attention should be paid to the management of overwintered *C. melanoneura* feeding on apple trees as soon as they return to apple orchards at the end of the winter. This is because they are the specimens with the higher phytoplasma load and thus the most hazardous stage for the spreading of the disease, promoting the long distance dissemination of the pathogen [19]. At the same time, based on the phytoplasma load of 3^rd^–5^th^ nymphs and newly emerged adults, we have proved the infective risk of these stages, which could cause the spreading of the disease within orchards, but this is more likely to occur in special situations, such as in the case of transovarial transmission of the phytoplasma in *C. melanoneura,* in orchards with a high infection rate and when the migration to the conifers is delayed.

The open question of possible phytoplasma transovarial transmission in *C. melanoneura* requires further investigation, because it could explain some of the obtained results and eliminate some remaining doubts. The vertical transfer of the phytoplasma to the progeny has been proved for the congeneric species *C. pruni* [36] and *C. picta* [37], and there is no reason to believe that this is not possible for *C. melanoneura*. This is considering the fact that the phytoplasmas hosted and transmitted by the three psyllid species belong to the same taxonomic group.

The obtained results refer to a specific geographical area (Aosta Valley, Northwest Italy) where *C. melanoneura* has been proved to be the main vector of ‘*Ca*. P. mali’, and are not necessarily transposable to other areas where even slight variations in climatic conditions, or different psyllid populations and phytoplasma strains could lead to different interplays between the pathogens and the insect vectors.

## 5. Conclusions

The ‘*Ca.* P. mali’ load in the psyllid vector *C. melanoneura* was monitored in all the life stages and throughout the year after controlled acquisition trials. We were able to detect the phytoplasma in all stages, highlighting a higher acquisition efficiency in the case of nymphs and newly emerged adults according to the time spent on the infected plant. The phytoplasma titer increased during the period spent on conifers for aestivation and overwintering, demonstrating a long latency period. Overwintered adults retained high quantities of phytoplasmas, representing the most worrisome stage. However, in several nymphs and newly emerged adults it was possible to detect a sufficient quantity of phytoplasmas to consider these stages infective. Therefore, in the investigated area, newly emerged adults represent a risk for the spread of the disease, particularly when the migration to the conifers is delayed for some reason.

## Figures and Tables

**Table 1:** Infection rate of *C. melanoneura* infected by ‘*Ca*. Phytoplasma mali’, phytoplasma titer (mean ± SE) and phytoplasma titer range observed in the acquisition study trials. AAP = acquisition access period; APP = apple proliferation phytoplasma.

Life Stage	AAP (days)	Total Tested Individuals (N.)	APP Infected Individuals (N.)	Infection Rate (%)	Phytoplasma Titer (APP GU/pg of Insect DNA)	Phytoplasma Titer Range (APP GU/pg of Insect DNA)
Overwintered adult naturally infected	-	180	3	1.67	2.04 × 10^3^ ± 5.44 × 10^2^	1.10 × 10^3^–2.98 × 10^3^
Overwintered adult	1	77	0	0.00	n.d.	n.d.
	2	80	1	1.25	4.39 × 10^2^ ± 0.00	4.39 × 10^2^
	10	91	6	6.59	2.27 × 10^3^ ± 1.12 × 10^3^	5.80 × 10–7.65 × 10^3^
	20	83	1	1.20	2.45 × 10^2^ ± 0.00	2.45 × 10^2^
Nymph (1^st^–2^nd^ instar)	10	42	3	7.14	4.29 ± 2.21	0.66–8.29
Nymph (3^rd^–5^th^ instar)	20	60	29	48.33	2.06 × 10 ± 1.22 × 10	0.01–3.52 × 10^2^
Newly emerged adult	30	83	35	42.17	3.17 × 10^2^ ± 8.22 × 10	0.09–1.74 × 10^3^

n.d.: no data available.

**Table 2:** Infection rate of newly emerged adults of *C. melanoneura* infected by ‘*Ca*. Phytoplasma mali’ and phytoplasma titer range after different days spent on conifers. APP = apple proliferation phytoplasma.

Days on Conifers	Total Tested Individuals (N.)	APP Infected Individuals (N.)	Infection Rate (%)	Phytoplasma Titer Range (APP GU/pg of Insect DNA)
5	5	0	0.00	n.d.
5–10	32	20	62.50	0.14–9.34 × 10^2^
10–15	90	28	31.11	0.17–1.23 × 10^3^
15–20	32	20	62.50	5.47–5.93 × 10^2^
20–30	103	30	29.13	0.17–2.24 × 10^3^
30–60	284	58	20.42	0.18–9.96 × 10^3^
60–90	35	11	31.43	5.74 × 10–1.81 × 10^4^
90–150	70	24	34.29	3.83 × 10^2^–1.35 × 10^4^
150–250	38	20	52.63	4.81 × 10^2^–1.42 × 10^4^
>250	65	43	66.15	1.13 × 10^2^–2.42 × 10^4^

n.d.: no data available.
